# Skin as an autonomous immune organ: Antibody production and host protection

**DOI:** 10.1016/j.apsb.2025.03.027

**Published:** 2025-03-13

**Authors:** Wende Deng, Ting Li

**Affiliations:** a State Key Laboratory of Quality Research in Chinese Medicines & School of Pharmacy, Faculty of Medicine, Macau University of Science and Technology, Macau 999078, China; b Macau Institute for Applied Research in Medicine and Health, Macau University of Science and Technology, Macau 999078, China; c Macau Institute for Artificial Intelligence in Medicine, Macau University of Science and Technology, Macau 999078, China

**Keywords:** Autonomous immune organ, Antibodies, Microbiota, Skin, Host protection

In a landmark study published in *Nature*^1^, Gribonika et al. redefine the role of the skin, revealing its ability to function as an “autonomous immune organ”. This discovery challenges the long-standing view of the skin as a passive physical barrier, demonstrating instead that the skin can independently regulate host–microbiota interactions. Notably, the study identifies the skin as a site for localized production of antibody subclasses, IgG2b and IgG2c, in response to common skin commensals like *Staphylococcus epidermidis* (*S. epidermidis*). These antibodies not only control bacterial growth locally but also provide systemic immune protection, underscoring the skin’s dual role in barrier defense and active immune regulation (Fig. 1).

**Figure 1:**
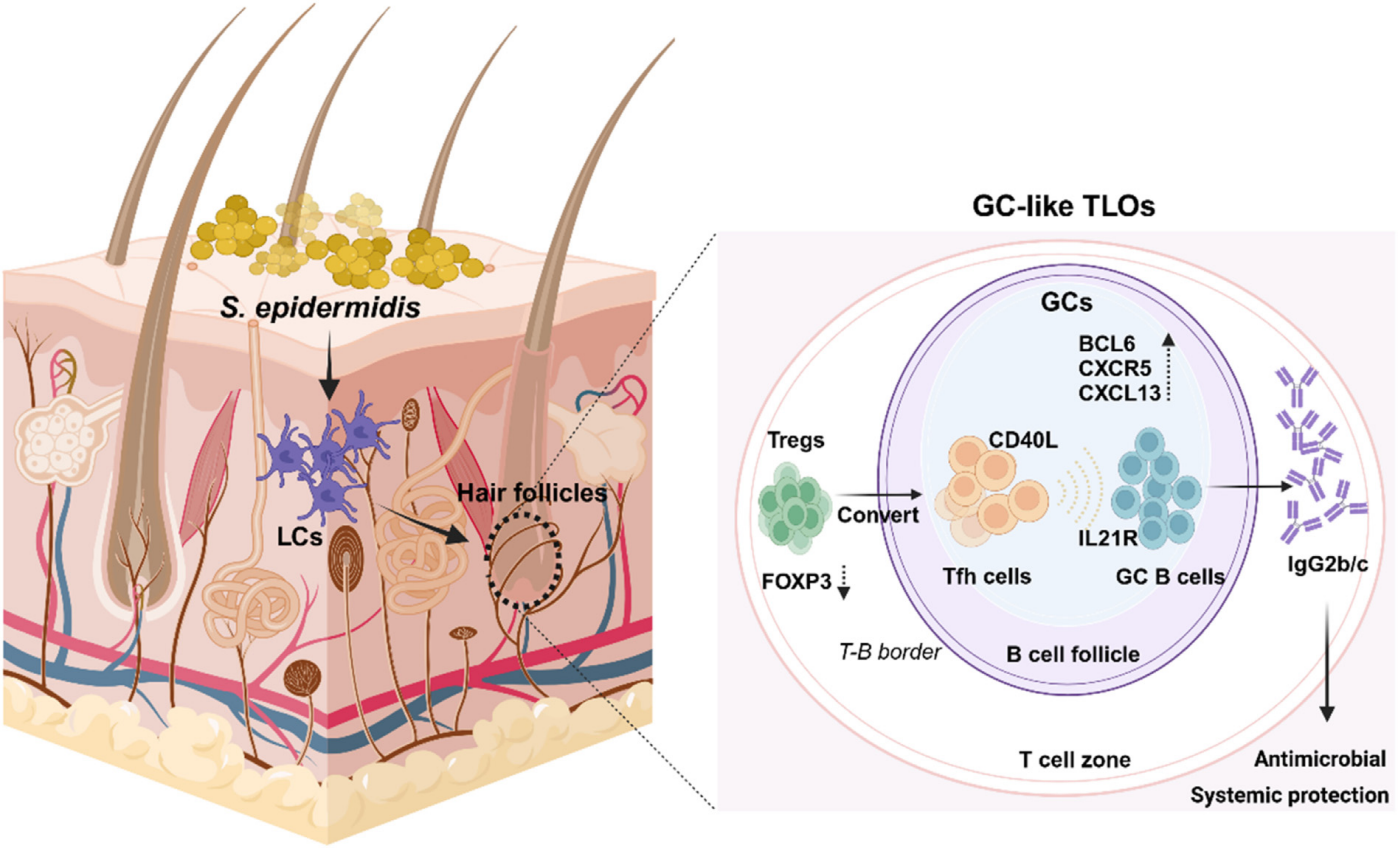
Mechanisms of skin autonomic immune response. *S. epidermidis* colonization can induce the formation of tertiary lymphoid organs (TLOs) around skin hair follicles. Langerhans cells (LCs) mediate this response by capturing and presenting antigens. In TLOs, LCs promote the conversion of regulatory T cells (Tregs) into T follicular helper (Tfh) cells, which are essential for germinal center (GC) formation. Key molecules such as BCL6, CXCL13, and CXCR5 play critical roles in regulating GC formation and recruiting both B and T cells to these sites. The converted Tfh cells then stimulate B cells to produce specific antibodies, especially IgG2b and IgG2c. These antibodies locally suppress bacterial growth and provide systemic protection through humoral immune responses, thereby maintaining skin microbiota homeostasis and preventing infections. (Created in BioRender. WD, D. (2025) https://BioRender.com/f31z283).

Historically, immune defense was attributed to secondary lymphoid organs (*e.g.*, lymph nodes), while the skin was perceived as lacking secondary lymphoid organs and incapable of generating mature B cells or functional antibody responses. However, emerging evidence, including Gribonika et al.’s findings, positions the skin as a dynamic interface with its microbiome. The skin microbiome, comprising bacteria, fungi, and viruses, is essential for maintaining cutaneous health. This symbiotic relationship is delicately balanced: barrier disruption or immune dysregulation can transform commensals into pathogens, triggering local infections or systemic diseases^2^. Beyond local effects, skin perturbations exert systemic influence. For example, skin injury induces nociceptive neuropeptide P release and mast cell degranulation^3^, while IL-33 signaling from damaged skin promotes intestinal ILC2 and mast cell activation, exacerbating food allergies^4^. Chronic skin inflammation can even lead to bone loss by inhibiting Wnt signaling in osteoblasts^5^. Collectively, these findings establish the skin as a central hub for systemic immune regulation, maintaining homeostasis and preventing infections.

*S. epidermidis*, long regarded as a passive resident of the skin, actively contributes to the immune regulation and skin homeostasis^6^. Gribonika et al. observed that colonization of *S. epidermidis* in specific pathogen-free (SPF) mice led to specific IgG production within two weeks, persisting for over 200 days. IgG2b emerged first, followed by IgG1 and IgG2c by Day 45. Compared to IgG1, *S. epidermidis*-specific IgG2b and IgG2c more potently activate the classical complement pathway and bind to cellular Fc receptors to generate opsonization effect and antibody-dependent cellular cytotoxicity (ADCC)^1^^,^^7^. These insights revolutionize the therapeutic concept of the skin in humoral immunity, offering a platform for development of safer and more effective vaccines.

The production of *S. epidermidis*-specific IgG occurs independently of secondary lymphoid organs, aligning with the progressive accumulation of B cells and the concurrent formation of tertiary lymphoid organs (TLOs) in the skin. TLOs, which harbor germinal centers (GCs), T-cell zones, and PANd^+^ high endothelial venules (HEVs), are organized lymphocyte aggregates formed during chronic inflammation of skin to regulate local immunity^8^. Their formation requires B cells, T follicular helper (Tfh) cells, and GCs, driven by molecules such as BCL6, CXCL13, and CXCR5. The function of the TLOs depends on IL-21R, CD40L, or Tfh cells. *S. epidermidis* colonization induces TLO formation around hair follicles, which serve as the primary site of microbial antigen capture and presentation, contributing to antibody response. These findings fill a critical gap by demonstrating that *S. epidermidis* colonization can induce TLO formation under physiological conditions, triggering humoral immune responses.

The skin-mediated immune response, however, relies heavily on Langerhans cells (LCs), the most abundant antigen presenting cells in the skin^9^. A key finding of this study is that LCs depletion significantly reduces the induction of Tfh cells and completely blocks the IgG response to *S. epidermidis*, reinforcing their essential role in Tfh responses and microbiota-driven humoral immunity in the skin. Fate mapping strategy revealed that >90% of symbiotic-reactive Tfh cells originate from FOXP3-deficient regulatory T cells (Tregs) following skin colonization with microorganism, suggesting that skin colonization induces the Tregs transdifferentiating into Tfh cells promoting the peripheral antibody response. By mounting a protective antibody response, mice with prior *S. epidermidis* colonization exhibit lower bacterial loads, whereas those without colonization develop systemic bacterial infections. Hence, colonization-induced pre-existing humoral immunity is the primary factor determining host protection, both in terms of physiology and infection.

By using *Bcl6*^*flox*^*Cd4*^*cre*^, *Cd4*0lg^–/–^, *Ltα*^–^^/^^–^ mice, the study further provides evidence that IgG antibodies produced exclusively in the skin are critical for controlling microbial load and preventing dysbiosis-driven inflammation. Therefore, the autonomous production of antibodies in the skin regulates host-microbiota interactions within the skin compartment and provides effective protection against subsequent infections. These findings highlight the crucial role of the skin in developing therapeutic strategy to treat chronic inflammation and systemic immune disorders.

Due to the critical role of commensals in the generation of local and systemic humoral immunity, they may advance cancer immunotherapy. By engineering *S. epidermidis* to express tumor antigens, combined with checkpoint inhibitors, researchers could ignite potent anti-tumor immunity^10^. Such microbial-based therapies promise targeted, low-toxicity alternatives to conventional cancer treatments.

In conclusion, Gribonika et al.’s work reshapes the unique role of the skin and its colonizing microbiota in humoral immunity, challenging the traditional view of the skin as a passive barrier. Their study elucidates how skin-microbiota interactions regulate humoral immunity, with implications for treating autoimmune diseases, infections, and cancer. Future studies exploring skin-immune crosstalk *via* antibody production, microbiota modulation, or metabolite regulation may yield transformative breakthroughs. Additionally, this paradigm shift highlights the potential of cutaneous vaccines to stimulate dual local and systemic immunity, enhancing protection against pathogens and sustaining immune homeostasis.

## Author contributions

Wende Deng: Writing - original draft, Conceptualization. Ting Li: Writing - review & editing, Conceptualization, Funding acquisition.

## Conflicts of interest

The authors declare no competing interests.
